# Gal*f*-Specific Neolectins: Towards Promising Diagnostic Tools

**DOI:** 10.3390/ijms25094826

**Published:** 2024-04-28

**Authors:** Mateja Seničar, Benoît Roubinet, Pierre Lafite, Laurent Legentil, Vincent Ferrières, Ludovic Landemarre, Richard Daniellou

**Affiliations:** 1 ICOA UMR CRNS 7311, Universite d’Orléans, Rue de Chartres, BP 6759, 45067 Orléans Cedex 2, France; mateja.senicar@univ-orleans.fr (M.S.); pierre.lafite@univ-orleans.fr (P.L.); 2 GLYcoDiag, 2 Rue du Cristal, 45100 Orléans, France; broubinet@glycodiag.com (B.R.); landemarre@glycodiag.com (L.L.); 3 Université de Rennes, École Nationale Supérieure de Chimie de Rennes, CNRS, ISCR, UMR 6226, 35000 Rennes, France; laurent.legentil@ensc-rennes.fr (L.L.); vincent.ferrieres@ensc-rennes.fr (V.F.); 4 Chaire de Cosmétologie, AgroParisTech, 10 Rue Léonard de Vinci, 45100 Orléans, France; 5 Université Paris-Saclay, INRAE, AgroParisTech, UMR Micalis, 78350 Jouy-en-Josas, France

**Keywords:** galactofuranosidase, neolectin bioengineering, site-directed mutagenesis, neoglycoprotein

## Abstract

In the absence of naturally available galactofuranose-specific lectin, we report herein the bioengineering of Gal*f*NeoLect, from the first cloned wild-type galactofuranosidase (*Streptomyces* sp. strain JHA19), which recognises and binds a single monosaccharide that is only related to nonmammalian species, usually pathogenic microorganisms. We kinetically characterised the Gal*f*NeoLect to confirm attenuation of hydrolytic activity and used competitive inhibition assay, with close structural analogues of Gal*f*, to show that it conserved interaction with its original substrate. We synthetised the bovine serum albumin-based neoglycoprotein (Gal*f*NGP), carrying the multivalent Gal*f* units, as a suitable ligand and high-avidity system for the recognition of Gal*f*NeoLect which we successfully tested directly with the galactomannan spores of *Aspergillus brasiliensis* (ATCC 16404). Altogether, our results indicate that Gal*f*NeoLect has the necessary versatility and plasticity to be used in both research and diagnostic lectin-based applications.

## 1. Introduction

Glycans, oligosaccharide moieties that originate from a small set of monosaccharides, are typically found conjugated to glycoproteins and glycolipids and are exposed to the extracellular side of membranes, where they mediate a tremendous variety of cell interactions and signalling [1,2,3]. Among the putative set of monosaccharide constituents of glycans in organisms, galactofuranose (Gal*f*), the five-membered ring form of aldohexose galactose, is completely absent from mammalian glycan motifs, including humans. Galactofuranose is by far the most widespread in other domains of life, ranging from archaea and bacteria to protozoa, fungi, and plants [4,5,6,7,8]. Often found in pathogenic organisms such as the bacterium *Mycobacterium tuberculosis*, the fungus *Aspergillus fumigatus*, and the protozoan *Leishmania major* as examples, it is hypothesised to be an advantageous element for their survival, reproduction, and virulence in the host [9]. As such, Gal*f*–deficient mutants in *Aspergillus* have significant modifications of the cell surface that result in aberrant morphological changes and growth reduction, whilst the virulence capacity of *Leishmania* species is reduced [10]. Therefore, exploiting the presence of Gal*f* in the glycome of pathogenic microorganisms makes it a promising target for biomedical research since it circumvents the potential risk of interference with the human glycans [11]. The distinctiveness of Gal*f* as a component of important surface glycoconjugates of human pathogens has led to increased interest in targeting it in diagnostic or imaging techniques. Recent diagnostic techniques are predominantly based on production of Gal*f*-specific antibodies. The presence of *Aspergillus* exoantigens of galactomannan (GM) origin is a specific indicator of invasive pulmonary aspergillosis [12]. Monoclonal antibody detection methods for early serological diagnosis of GM antigens of invasive pulmonary aspergillosis have been experimentally developed since the 1980s. In 1995, a double-direct sandwich enzyme-linked immunosorbent assay (ELISA), that uses a rat anti-GM monoclonal antibody (EB-A2) directed against the β-Gal*f*-(1 → 3)-β-Gal*f* epitopes of GM, was developed. Today, two GM antigen-detection kits are commercially available, the ELISA Pastorex^®^ *Aspergillus* test (Sanofi Diagnostics Pasteur, Marnes-La-Coquette, France) and the latex agglutination test (LAT) Platelia^™^ *Aspergillus* (Biorad, Marnes-la-Coquette, France) [10]. The bibliographical overview of different types of Gal*f*-specific antibodies is summarised in Table 1.

In addition to Gal*f*-specific antibodies, there is another class of carbohydrate-binding proteins of nonimmune origin, lectins, that can be used as tools for detecting Gal*f*-bearing motifs. Unfortunately, only a few natural lectins have been characterised so far as Gal*f*-binding and have mostly been used in research studies, among which human intelectin-1 (hIntL-1) has been closely studied [32,33,34,35]. In addition, all of them do not specifically recognise Gal*f*, but also multiple glycan epitopes engaged in host–pathogen recognition interactions (Table 2). We focus herein on another type of Gal*f*-recognising lectin, neolectin, bioengineered from naturally occuring Gal*f*-ase. First described in 1977 and purified in crude form from the culture supernatants and cell lysates of filamentous fungi, bacteria, and protozoa, it was only in 2015 that an open reading frame (ORF) encoding a Gal*f*-specific enzyme from Gram-positive bacteria *Streptomyces* sp. (strain JHA19) was identified, cloned, expressed, and completely biochemically characterised [36,37,38,39,40]. 

**Figure 1:**
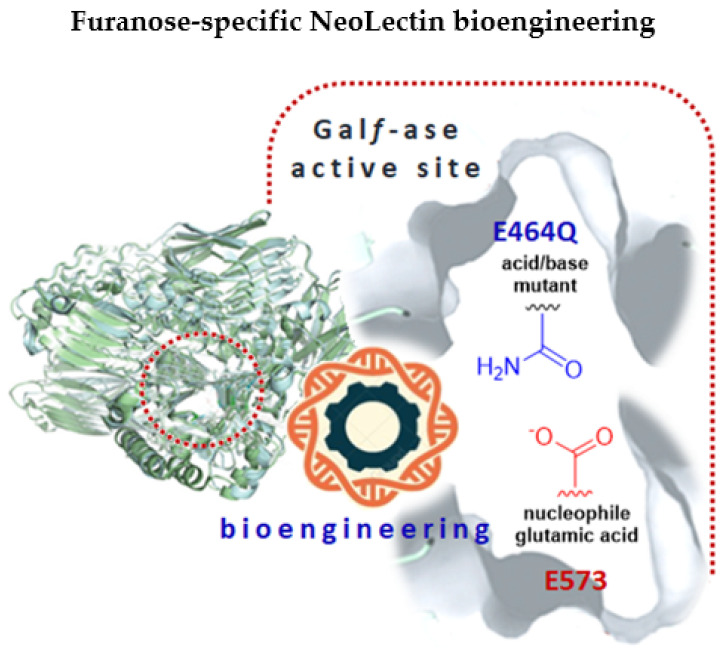
A graphical abstract depicting the bioengineering of NeoLectins from galactofuranosidase by the site-directed mutagenesis method. The glutamic acid/base catalytic residue (E464) was specifically changed to glutamine (Q), resulting in galactofuranosidase E464Q mutant with attenuated hydrolytic activity that can act as galactofuranose-binding neolectin (Gal*f*NeoLect).

In this work, we have bioengineered, from Gal*f*-specific carbohydrate-processing enzyme, through site-directed mutagenesis, an inactivated Gal*f*-binding neolectin (Gal*f*NeoLect) (Figure 1) [44]. As a probe to investigate and validate its binding specificity, we synthesised the bovine serum albumin (BSA)-based neoglycoprotein carrying the Gal*f*, called Ga*lf*NGP. Using an integrated approach that combined enzyme kinetic assays, inhibition assays, and GLYctPROFILE^®^ assays, we validated the Gal*f*NGP and Gal*f*NeoLect systems for their use to determine and study the Gal*f*-specific recognition and binding.

## 2. Results

### 2.1. Design and Production of Novel Galf-Specific Neolectin (GalfNeoLect)

**Figure 2:**
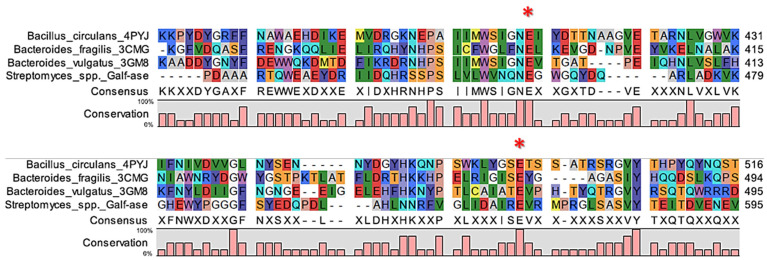
Alignment of partial ORF of Gal*f*-ase (*Streptomyces* spp.) amino acid sequence and corresponding regions of its homologs. The conserved E464 and E573 amino acid residues are indicated with asterisks.

Our strategy was to generate several mutant variants of the wild-type Gal*f*-ase characterised by attenuated hydrolytic activity towards the *p*NP β-D-Gal*f* substrate. Since the deduced Gal*f*-ase amino acid sequence analysis, as previously reported [38,39,40,45], revealed that it belongs to the GH2 family, to identify the catalytic residues of interest, we carried out in silico sequence alignment on three bacterial β-galactosidases from the GH2 family with determined crystal structures. The selected homologous amino acid sequences from *Bacillus circulans* (PDB accession code 4YPJ), *Bacteroides fragilis* (PDB accession code 3CMG), and *Bacteroides vulgatus* (PDB accession code 3GM8), sharing between 24% and 26% of their identities, revealed two sets of absolutely conserved glutamic acid residues (Figure 2, Supplemental Figure S3). 

**Figure 3:**
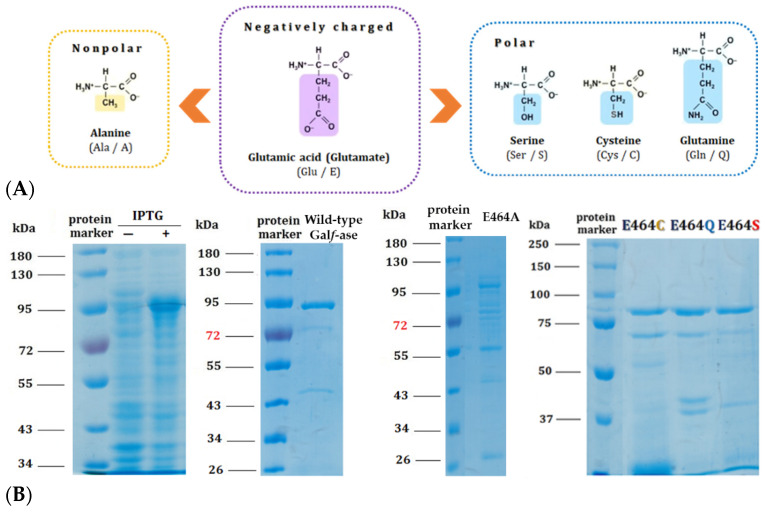
(**A**) Structural formulas of amino acids selected for the generation of E464X Gal*f*-ase mutant variants. (**B**) SDS–PAGE analysis of wild-type Gal*f*-ase after IPTG induction and Ni-NTA chromatography purification, as well as Gal*f*-ase E464A, E464C, E464Q, and E464S mutants. Lane 1: protein marker. Lanes 2 and 3: cells’ extract before and after IPTG’s induction of wild-type Gal*f*-ase. Lane 4: protein marker. Lane 5: purified wild-type Gal*f*-ase. Lane 6: protein marker. Lane 7: traces of E464A mutant (not obtained in purified form). Lane 8: protein marker. Lane 9 to 11: purified E464C, E464Q, and E464S mutants.

Based on mechanistic grounds of the GH2 family, the conserved residues represent the catalytic nucleophile (E573) and the catalytic acid/base (E464). Identified glutamic acid residues were expected to be the catalytic ones [40] and, therefore, we focused our mutagenesis studies on the acid/base glutamic acid residue (E464) that has a crucial step in glycohydrolitic catalysis. Therefore, the acid/base catalytic residue (E464) has been specifically substituted to alanine (E464A), serine (E464S), cysteine (E464C), and glutamine (E464Q) (Table 3). The acid/base carboxylic acid residue (E464) has been replaced by the amino acids that have the residues of specific interest—briefly, that have no negative charge—and, subsequently, the hydrolytic mechanism rate should be attenuated whilst conserving the substrate specificity, in order to generate and explore their potential role as Gal*f*-specific neolectins (Figure 3A). All of the Gal*f*-ase mutant protein variants, except E464A, were produced and purified under the same conditions as wild-type Gal*f*-ase. It appears that the E464A mutation probably affected the folding and stability of protein, resulting in the formation of insoluble protein aggregates (Figure 3B).

### 2.2. Kinetic Characterisation of Galf-Ase Mutant Variants—Identification of Novel Galf-Specific Neolectin (GalfNeoLect)

In analysing the hydrolitic activity and the hydrolysis rate-limiting steps of the three Gal*f*-ase mutant variants E464S, E464C, and E464Q, the Michaelis–Menten kinetic parameters were determined under the similar conditions used for wild-type enzyme [46]. All the mutants gave typical Michaelis–Menten saturation curves (Supplemental Figure S4) and exhibited slightly decreased *K*_M_ values compared to the one determined for wild-type, apart from E464S, which stands out for its sevenfold decrease in the *K*_M_ value (Table 4). Altogether, the obtained values are in the same order of magnitude. Indeed, the replacement of glutamic acid residue to serine, cysteine, and glutamine has resulted in a 2000-orders-of-magnitude decrease in the hydrolytic activity compared to the wild-type Gal*f*-ase enzyme, confirming that this residue is essential for catalysis. The significant decrease in catalytic efficiency by a factor of 3000–1400 times for E464S and E464C mutants, respectively, and 56 times lower for E464Q mutant is observed. These kinetic observations are in accordance with the other data obtained in similar assays used for the identification of the catalytic residues in retaining and inverting several GHs [46,47,48]. The Gal*f*-ase mutant variant E464Q featured only a minor change in the *K*_M_ value, 166 µM compared to 250 µM, of the wild-type Gal*f*-ase and, given the advantage that, contrary to other mutant variants, it was purified in sufficient quantities (0.3 mg/L), it was selected as a promising candidate to be tested as a novel Gal*f*-binding lectin (Gal*f*NeoLect). Further in the article, the E464Q mutant variant is addressed as Gal*f*NeoLect.

### 2.3. Galf-Conjugated BSA Neoglycoprotein (GalfNGP) as Novel Ligand for GalfNeoLect

**Figure 4:**
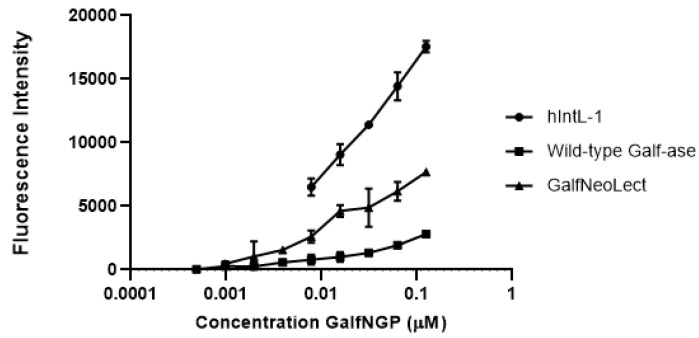
Binding interaction of biotinylated Gal*f*NGP to immobilised hIntL-1, wild-type Gal*f*-ase, and Gal*f*NeoLect. Gal*f*-ase E464Q mutant variant was selected as a novel Gal*f*-binding lectin (Gal*f*NeoLect).

To study and evaluate the binding interactions of Gal*f*-ase E464Q mutant variant, as novel Gal*f*-binding lectin (Gal*f*NeoLect), we synthetised novel neoglycoproteins (NGPs) to serve as the Gal*f*-bearing scaffold. Bovine serum albumin (BSA), a well-known, naturally unglycosylated protein that has up to 60 primary amino functional groups available for click-conjugation chemistry [49], has been functionalised with Gal*f* motifs. Gal*f*-N_3_, involved in the corresponding click reaction with propargyl functionalised BSA, was obtained from the corresponding per-*O*-acetyl galactofuranose [50] using TMSN_3_ as a source of azide and TMSOTf as the Lewis acid (see ESI for chemical synthesis). After acetyl cleavage, the resulting/expected Gal*f*-conjugated BSA neoglycoprotein (Gal*f*NGP) synthesis has been first characterised for its functionality by direct binding assay with human Intelectin-1 (hIntL-1), wild-type Gal*f*-ase, and Gal*f*NeoLect. As hIntL-1 has been reported to bind furanose residues (five-membered-ring saccharide isomers), including a β-Gal*f*–containing disaccharide, it served as a positive control for the evaluation of newly synthetised Gal*f*NGP. The binding ability towards Gal*f*NGP was tested directly by an enzyme-linked lectin assay (ELLA), where the immobilised hIntL-1, wild-type Gal*f*-ase, and Gal*f*NeoLect were incubated in the presence of increasing concentrations of Gal*f*NGP. Under these conditions, the all three tested proteins gave dose-dependent responses (Figure 4) towards Gal*f*NGP with BC_50_ (concentration to obtain 50% Gal*f*NGP binding with the selected proteins) values in the same range of magnitude (Table 5). Indeed, BC_50_ values at 0.16 μM for Gal*f*NeoLect and hIntL-1 were obtained. These values are comparable and in accordance with the 0.085 ± 0.014 μM functional affinity value obtained with β-D-Gal*f*-substituted surface for hIntL-1 in a previous study [33]. These results demonstrate that Gal*f*NGP is a novel and relevant ligand for testing the binding recognition towards Gal*f.*

In parallel, hIntL-1, wild-type Gal*f*-ase, and Gal*f*NeoLect were tested in direct binding assay with standard NGPs that carry out the following carbohydrates: α-D-Galactose, α-L-Fucose, α-D-Mannose, and D-Glucose, as negative controls, which have not shown binding properties towards hIntL-1, wild-type Gal*f*-ase, and Gal*f*NeoLect (Supplemental Table S1).

### 2.4. Profiling GalfNeoLect Specificity 

**Figure 5:**
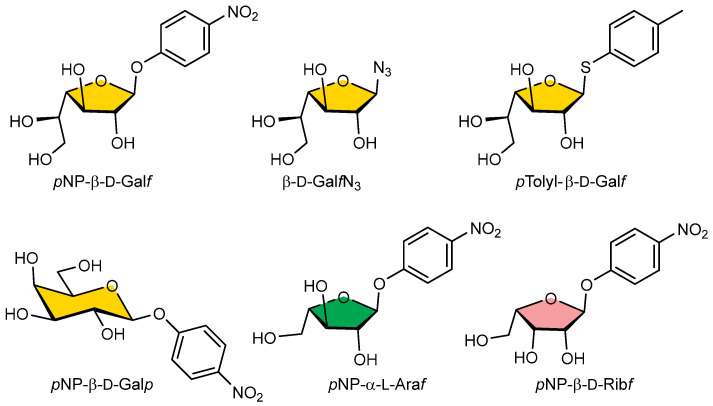
Structural formulas of *p*NP-pyranosyl, -furanosyl, azido, or thioaryl substrates used for wild-type Gal*f*-ase and Gal*f*NeoLect substrate specificity screening.

In the competitive inhibition assay, we investigated the ability of selected *p*NP, azido, or thyoaryl monosaccharide substrates (Gal*f*-N_3_, Gal*f*-thioaryl, *p*NP-β-D-Gal*f*, *p*NP-β-D-Gal*p*, *p*NP-α-L-Ara*f*, *p*NP-β-D-Rib*f*) (Figure 5) for their ability to inhibit the binding of Gal*f*NGP to immobilised Gal*f*NeoLect.

The presence of increasing concentrations of Gal*f*-N_3_, Gal*f*-thioaryl, and *p*NP-β-D-Gal during incubation clearly resulted in a progressive decrease in the fluorescence signals for Gal*f*NeoLect in a dose-dependent manner (Supplemental Figure S5). Indeed, Table 6 summarises the 50% inhibitory concentrations (IC_50_) obtained from all compounds studied. The lowest IC_50_ was obtained for Gal*f*-thioaryl with 0.18 mM, followed by Gal*f*-N_3_ with 0.60 mM. On the contrary, feeble responses in terms of detectability and no inhibition were obtained with *p*NP-β-D-Gal*p*, *p*NP-α-L-Ara*f*, and *p*NP-β-D-Rib*f* monosaccharide substrates (Supplemental Figure S5, and Table 6), which demonstrate their absence of specificity for the tested Gal*f*NeoLectin. These results are in accordance with the IC_50_ values (Table 6, Supplemental Figure S6) obtained in the competitive inhibition assay with the wild-type Gal*f*-ase, which interacts with the identical type of inhibitors (Gal*f*-thioaryl) and in the same range of values as shown in a previous study [46]. These results demonstrate that the Gal*f*NeoLectin binds specifically to only Gal*f*-motifs and that the site-directed mutagenesis did not affect the substrate specificity. 

**Figure 6:**
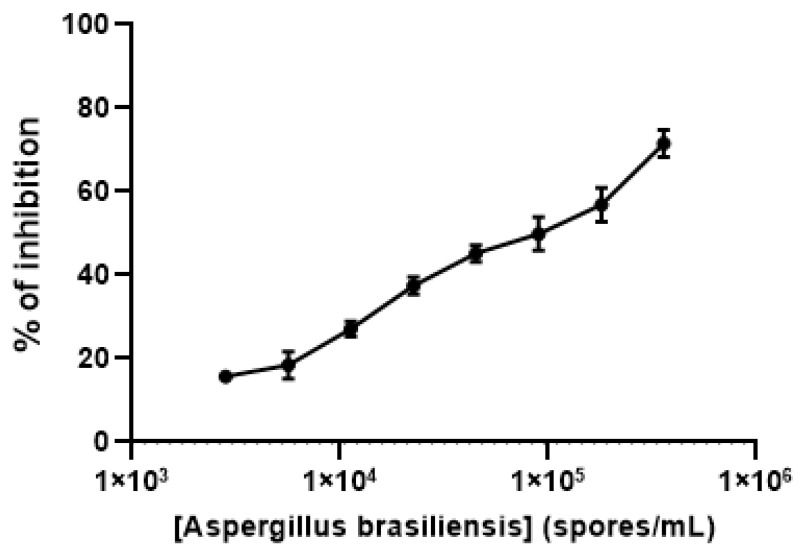
Inhibition profile of *Aspergillus brasiliensis* (ATCC 16404) spores with Gal*f*NeoLect. Biotinylated Gal*f*NGP (C = 2 μg/mL) was used as a tracer. Briefly, the *A. brasiliensis* (ATCC 16404) spores naturally carry on its surface Gal*f*-containing galactomannan which is in competition for binding to Gal*f*NeoLect with biotinylated Gal*f*NGP.

Finally, to test the biological functionality of the Gal*f*NeoLect on crude samples, we used the viable spores from *Aspergillus brasiliensis* (ATCC 16404) that naturally carry Gal*f*-containing galactomannan. By inhibition assay, we demonstrate that *Aspergillus* spores are able to displace the interaction between Gal*f*NGP and Gal*f*NeoLect in a dose-dependent manner (Figure 6). 

## 3. Discussion

To bioengineer a galactofuranose-specific neolectin (Gal*f*NeoLect) from a glycoside hydrolase, galactofuranosidase (Gal*f*-ase), we undertook several steps, each followed by a qualitative procedure to determine the following parameters: (a) inactivation rate of hydrolytic activity, (b) conservation of substrate specificity, and (c) probing a robustness Gal*f*-recognition capacity on a novel substrate. In the lack of the available resolved Gal*f*-ase crystal structure, the prediction of the active site amino acid residues was carried out by sequence comparison with the enzymes within the same GH2 family. Based on the sequence identities between 24% and 26% with the *Bacillus circulans*, *Bacteroides fragilis*, and *Bacteroides vulgatus* GHs, two glutamic acid residues, E464 and E573, were highlighted as active site acid/base and nucleophile residues. In the present study, we used the site-directed mutagenesis method for direct confirmation of catalytic function of selected residues. The reduction in the catalytic activities of the mutant enzymes E464S, E464C, and E464Q was consistent with the suggested roles of the altered amino acids as catalytic residues. As indicated by kinetic studies, the catalytic activities of mutant enzymes (k_cat_/*K*_M_) were 3.000 to 56-fold weaker than that of the wild-type Gal*f*-ase. According to the performance of its kinetic parameters, on-hand quantities, and the fact that, as the amide analogue is substituted to glutamic acid, it does not vary the length of aliphatic chain, the E464Q mutant variant was considered to have satisfying qualities to act as Gal*f*NeoLect. We performed an assessment of whether the change in the catalytic acid/base residue had an impact on the substrate specificity of Gal*f*NeoLect, the variety of *p*NP, azido, or thyoaryl monosaccharide substrates (Gal*f*-N_3_, Gal*f*-thioaryl, *p*NP-β-D-Galf, *p*NP-β-D-Gal*p*, *p*NP-α-L-Ara*f*, *p*NP-β-D-Rib*f*), including α-L-Ara*f*, a stereochemical analogue of β-D-Gal*f*, differing only by the absence of the extra C-6 hydroxymethyl group. Only three classes of substrates, all having Gal*f*-sugar moiety, showed interaction with Gal*f*NeoLect, bare Gal*f*-N_3,_ *p*NP-β-D-Gal*f* and its thioaryl analogue of Gal*f*. Data from competitive inhibition assay reveal that Gal*f*NeoLect recognises multiple Gal*f*-bearing molecules and can discriminate them between other monosaccharides. Also, we synthetised a novel neoglycoprotein based on Gal*f*-decorated bovine serum albumin to investigate whether Gal*f*NeoLect interacts with the more complex structures. BSA has been widely used as scaffold for harbouring multiple carbohydrate motifs via click chemistry [49]. The resulting polyvalent Gal*f*NGP mimics carbohydrate presentation at the cell surface and allows one to study the Gal*f*NeoLect–carbohydrate interactions. The Gal*f*-binding capacity of Gal*f*NGP was investigated with hIntL-1, a galactofuranosyl-binding lectin that served as standard, and compared with wild-type Gal*f*-ase and Gal*f*NeoLect in the direct binding assay. The wild-type Gal*f*-ase and Gal*f*NeoLect, together with hIntL-1, showed binding to Gal*f*NGP, indicating that the Gal*f*-moieties are able to trigger the specific interactions with Gal*f*NGP.

## 4. Materials and Methods

### 4.1. Biological and Chemical Reagents

*E. coli* Rosetta™ (DE3) and plasmid vector pET-28a (+) were purchased from Novagen^®^. All *p*-nitrophenyl monosaccharides (*p*NP sugars) were purchased from Carbosynth (Compton, UK). All other laboratory chemicals used as the starting compounds, reagents, and solvents were analytical-grade purity and commercially available, unless otherwise specified. Neoglycoproteins (NGPs) used in direct binding assays were obtained from GLYcoDiag (Orléans, FRANCE): α-D-Galactose neoglycoprotein (NeoGa), α-L-Fucose neoglycoprotein (NeoF), α-D-Mannose neoglycoprotein (NeoM), and α-D-Glucose neoglycoprotein (NeoaG). *Aspergillus brasiliensis* (ATCC 16404) was obtained from an American-type culture collection (Manassas, VA, USA).

### 4.2. In Silico Sequence Analysis

Gal*f*-ase nucleotide and amino acid sequences were retrieved from the National Centre for Biotechnology Information (NCBI) GenBank^®^ (http://www.ncbi.nlm.nih.gov/genbank/, accessed on 22 July 2019) database under accession number LC073694. Amino acid sequence alignment search was conducted using a protein query BLAST^®^ (Basic Local Alignment Search Tool) (http://blast.ncbi.nlm.nih.gov/Blast.cgi, accessed on 22 July 2019) in the Protein Data Bank (PDB) database. The deduced protein amino acid sequences, deposited under PDB accession codes 4YPJ (*B. circulans*, 26% identities), 3CMG (*B. fragilis*, 24% identities), and 3GM8 (*B. vulgatus*, 25% identities), were aligned and analysed with CLC Sequence Viewer 7.7 software (Qiagen, Denmark).

### 4.3. Site-Directed Mutagenesis, Overexpression, and Mutant Galf-Ase Protein Purification

A pET-28a(+) plasmid construct encoding the wild-type galactofuranosidase (Gal*f*-ase) gene from JHA 19 *Streptomyces* sp. [46] was used as a template to generate the mutant Gal*f*-ase genes (E464Q, E464A, E464C, E464S) that were obtained by the site-directed mutagenesis method. The mutagenic primers (Table 7) were designed with Agilent Technologies QuikChange Primer Design tool and the reaction was performed using Agilent Technologies QuikChange Lightning Site-Directed Mutagenesis Kit (Santa Clara, CA, USA). The presence of desired mutations was confirmed by DNA sequencing performed by Eurofins Genomics. The overexpression and purification of mutant Gal*f*-ase proteins was performed following a procedure reported previously [46]. Briefly, *E. coli* Rosetta™ (DE3) (Novagen^®^) expression strain, transformed with the plasmid construct carrying the desired Gal*f*-ase mutant genes, was grown overnight at 37 °C in 1 L Luria–Bertani medium (LB media) supplemented with chloramphenicol (34 μg/mL) and kanamycin (30 μg/mL). Cells were grown to mid-exponential phase (OD_600_: 0.6), then shortly cooled on ice, and protein expression was induced by the addition of β-D-thiogalactopyranoside (IPTG) (100 μL; 1 M), after which the mixture was left on a shaker incubator (250 rpm) for 16 h at 15 °C. Afterwards, cells were harvested by centrifugation (4255× *g*, 30 min, 4 °C) and the cell pellets were resuspended in lysis buffer solution (1:10 *v*/*v*; 100 mM NaCl, 50 mM Tris/HCl pH 8, 1 mM phenylmethanesulfonyl fluoride (PMSF), 5% glycerol, 0.1% Triton X-100, lysozyme 1 mg/L). The suspension was incubated by stirring for 20 min at 4 °C, lysed by three freeze–thaw cycles, and subsequently sonicated on ice. Lysate was centrifuged (30,000 g, 20 min, 4 °C), supernatant was filtered (0.45 μm pore filter), and the protein was then purified from the clarified lysates using pre-equilibrated (10 mL; 50 mM Tris, 200 mM NaCl, 10 mM Imidazole) Thermo Scientific HisPur™ Ni-NTA Chromatography Cartridge (1 mL). The bound protein was eluted by an imidazole gradient (10–500 mM) and an aliquot of eluted fractions was analysed by sodium dodecyl sulfate–polyacrylamide gel electrophoresis (SDS–PAGE) on 8% separating gel, according to Laemmli’s method [51], and protein bands were visualised by staining with Coomassie Brilliant Blue G250. Fractions containing pure protein were collected and concentrated by ultrafiltration (30,000 MWCO, Sartorius Vivaspin^®^ sample concentrator), and protein quantity was determined using colorimetric Bio-Rad protein assay based on the Bradford dye-binding method, with bovine serum albumin (BSA) as a standard.

### 4.4. Kinetic Studies

Kinetic parameters were determined with *p*NP-β-D-Gal*f* as a substrate in different concentration ranges (0.01–5 mM). The reaction (200 μL), containing buffer (20 μL; 0.1 M citric acid/0.2 M Na_2_HPO_4_ pH 4.5) and water, if required, was started by addition of the enzyme (2 μL; 0.13 mg/mL) and, after 20 min of incubation at 37 °C (water bath), the reaction was terminated (100 μL; 1 M Na_2_CO_3_) and absorbance (405 nm) of released *p*NP was measured. All the reactions were assayed in triplicate and absorbance values were corrected for the spontaneous hydrolysis of the substrate. The kinetic parameters (*K*_M_, V_m_, k_cat_) were calculated using GraphPad Prism 5 software (GraphPad Software, San Diego, CA, USA).

### 4.5. General Procedure for the Preparation of β-d-Galactofuranosyl Azide (Galf-N_3_)

2,3,5,6-tetra-*O*-acetyl-β-d-galactofuranosyl azide: To a solution of per-*O*-acetylgalactofuranose [50] (500 mg, 1.28 mmol) in anhydrous dichloromethane (15 mL), we added trimethylsilyl azide (0.34 mL, 2.56 mmol) and trimethylsilyl trifluoromethanesulfonate (0.69 mL, 3.84 mmol), successively, at RT. After stirring for 3 h, Et_3_N (0.5 mL) was added and the solvent was evaporated under reduced pressure. The residue was purified by chromatography (cylohexane/EtOAc, 7:3) to give 2,3,5,6-tetra-*O*-acetylgalactofuranosyl azide (360 mg, 75%) as a mixture of anomers (β/α, 95:5 according to NMR spectra) and as a colourless oil. ^1^H NMR (β anomer, CDCl_3_): δ 5.49 (1H, s, H-1), 5.38 (1H, td, *J* = 6.8, 4.8 Hz, H-5), 5.06 (1H, ddd, *J* = 4.8, 1.7, 0.6 Hz, H-3), 4.95 (1H, dd, *J* = 2.0, 1.7 Hz, H-2), 4.38 (1H, t, *J* = 4.8 Hz, H-4), 4.35 (1H, dd, *J* = 12.0, 4.8 Hz, H-6), 4.21 (1H, dd, *J* = 12.0, 6.8 Hz, H-6′), 2.14 (3H, s, C*H_3_*CO), 2.13 (3H, s, C*H_3_*CO), 2.11 (3H, s, C*H_3_*CO), 2.07 (3H, s, C*H_3_*CO). ^13^C NMR (β anomer, CDCl_3_): δ 170.5, 170.0, 169.8, 169.5 (CO), 94.2 (C-1), 82.1 (C-4), 81.0 (C-2), 76.4 (C-3), 69.2 (C-5), 62.4 (C-6), 20.8, 20.7, 20.6 (*C*H_3_CO). HRMS (ESI): calcd for C_14_H_19_N_3_O_9_Na [M + Na]^+^ 396.1019, found 396.1018. Anal. Calcd for C_14_H_19_N_3_O_9_: C, 45.04; H, 5.13; N, 11.26. Found: C, 45.42; H, 5.27; N, 10.95.

β-d-galactofuranosyl azide (Gal*f*-N_3_): To a solution of 2,3,5,6-tetra-*O*-acetylgalactofuranosyl azide (300 mg, 0.81 mmol) in anhydrous methanol (8 mL), we added a solution of sodium methoxide in methanol (0.1 M, 80 μL, 0.08 mmol). The reaction mixture was stirred at room temperature for 12 h. Then, the solution was neutralised by addition of Amberlyst IR-120 H+ resin. After filtration, the filtrate was evaporated under vacuum and the residue was dissolved in water for freeze-drying to give targeted β-d-galactofuranosyl azide (164 mg, quantitative yield) as a white powder (Supplemental Figure S1). ^1^H NMR (400 MHz, D_2_O): δ 5.31 (1H, d, J = 2.8 Hz, H-1),4.06 (1H, dd, J= 10.4, 3.6 Hz, H-3), 4.01 (1H, dd, J = 10.4, 4.4 Hz, H-4), 3.94 (1H, dd, J = 3.6, 2.8 Hz, H-2), 3.80–3.73 (1H, m, H-5), 3.64 (1H, dd, J = 11.6, 4.8Hz, H-6), 3.57 (dd, J = 11.6, 7.2 Hz, 1H, H-6′); ^13^C NMR (100 MHz, D_2_O,): δ 95.3 (C-1), 84.2 (C-4), 80.6 (C-2), 76.4 (C-3), 70.7 (C-5), 62.6 (C-6). HRMS (ESI): calcd for C_6_H_11_N_3_O_5_Na [M + Na]^+^ 228.0596, found 228.0602.

### 4.6. Synthesis of Galf-Conjugated BSA Neoglycoprotein (GalfNGP)—Conjugation of BSA-Alkynewith Galf-N_3_

To a solution of BSA-alkyne [49] (10 mg, 10 mg/mL, 1 equiv.) in PBS (10 mM, pH 7.2), we added, successively, Gal*f*N_3_ (2 mg, 60 equiv. in solution at 10 mg/mL in PBS) L-ascorbic acid (0.5 mg, 21 equiv., in solution at 3 mg/mL in PBS), CuSO_4_·5H_2_O (0.4 mg, 10.5 equiv. in solution at 3 mg/mL in PBS), and tris[(1-benzyl-1*H*-1,2,3-triazol-4-yl)methyl]amine (TBTA, 2.6 mg, 35 equiv. in solution at 5 mg/mL in DMSO). The solution was stirred for 24 h at room temperature, then the crude mixture was purified by size-exclusion chromatography on Sephadex G25. The purity of Gal*f*NGP was controlled by SDS–PAGE and the number of Gal*f* motifs presented on BSA was determined by MALDI–TOF (UltrafleXtreme, Brucker) (Supplemental Figure S2). 

### 4.7. Direct Binding Assays

The assays were performed according to GlycoDiag’s protocol, already described in the literature [49,52,53,54,55]. Briefly, the neoglycoprotein preliminary, labelled with biotin (in a range of concentrations), was prepared in PBS supplemented with 1 mM CaCl_2_ and 0.5 mM MgCl_2_ and deposited in each well (50 μL each) of 96-well plates, previously functionalised with either Intelectin (hIntL-1, 9137-IN-050, Biotechne), wild-type Gal*f*-ase, or Gal*f*NeoLect (LEctPROFILE^®^ plates from GLYcoDiag, Orléans, France) in triplicate and incubated for 2 h at room temperature. After washing with PBS buffer, the streptavidin-DTAF conjugate was added (50 μL) and incubated for 30 min. The plate was then washed again with PBS. Finally, 100 μL of PBS was added for reading the plate using a fluorescence reader (λex = 485 nm, λem = 530 nm, Fluostar OPTIMA, BMG LABTECH, France). The intensity of the signal was directly correlated with the ability of the compound to be recognised by the lectin.

### 4.8. Inhibition Assays

The interaction profiles of each compound were determined through an indirect method, based on the inhibition by the compound of the interaction between a specific coupled lectin–glycan (a neoglycoprotein labelled with biotin and used as a tracer [48]). Briefly, a mix of biotinylated Gal*f*NGP (fixed concentration) and the corresponding compounds (in a range of concentrations), prepared in PBS supplemented with 1 mM CaCl_2_ and 0.5 mM MgCl_2_, are deposed in each well (50 μL each) in triplicate and incubated for 2 h at room temperature. After washing with PBS buffer, the conjugate streptavidin-DTAF was added (50 μL) and incubated for 30 min more. The plate was washed again with PBS. Finally, 100 μL of PBS was added for the readout of the fluorescent plate, performed with a fluorescence reader (λex = 485 nm, λem = 530 nm, Fluostar OPTIMA, BMG LABTECH, France). The signal intensity was inversely correlated with the capacity of the compound to be recognised by the lectin and expressed as inhibition percentage with comparison with the corresponding biotinylated Gal*f*NGP alone.

### 4.9. Preparation of Aspergillus Brasiliensis (ATCC 16404) Spores

*Aspergillus brasiliensis* (ATCC 16404) culture was inoculated on Sabouraud agar plates and incubated at 37 °C for 4 days until the formation of a black mycelium mat, which contains spores. Fresh spores were harvested with a sterile plastic inoculation loop by rubbing the black mycelium mat and dispersed in physiological serum solution (0.8% NaCl, 10 mL). The spore solution was resuspended well by using a vortex to prevent aggregation and was calibrated using a Malassez counting chamber to 5.10^6^ spores/mL. Spores’ solutions (5.10^6^ spores/mL) were used directly in inhibition assays with Gal*f*NeoLect, following the same protocol describe above (Section 4.8).

## 5. Conclusions

A natural lectin that specifically binds only a Gal*f* epitope and does not engage with other glycan epitopes has not yet been discovered. Therefore, to the best of our knowledge, we generated the first Gal*f*-specific neolectin from the corresponding wild-type galactofuranosidase that exclusively recognises and interacts with Gal*f*-glycan motifs. Furthermore, we synthetised the novel neoglycoprotein-carrying Gal*f* monosaccharide units and demonstrated that it is recognised (in this study) by all available Gal*f*-binding, hIntL-1, and Gal*f*-specific proteins, as well as wild-type Galf-ase and Gal*f*NeoLect. Furthermore, it may be feasible to exploit the Gal*f* multivalency of Gal*f*-NGP to identify enzymes involved in Gal*f* metabolism and, together with Gal*f*NeoLect, it is an interesting complementary Gal*f*-oriented system to study host–pathogen interactions or for the qualitative GLYcoPROFILE^®^ serodiagnosis of infectious diseases such as aspergillosis, leishmaniasis, borreliosis (Lyme disease), or tuberculosis.

## 6. Patents

Daniellou, R.; Lafite, P.; Seničar, M.; Roubinet, B.; Landemarre, L.; Néolectine spécifique et son utilisation pour détecter des microorganismes.
**Patent No.****Kind****Date****Application N°****Date**FR3116062A113 May 2022FR20114446 November 2020WO2022096829A112 May 2022WO2021-FR519444 November 2021

## Figures and Tables

**Table 1:** Comparative summary of antibodies specific for different Gal*f*-containing glycan motifs.

	Antibodies				
Origin	Type	Epitope Specificity	Target Organism	Purpose	References
Human serum Ab/rabbit Ab	IgG	EPS/(1,5)-linked β-D-galactofuranosides	*Penicillium* & *Aspergillus* species	research study	[13,14]
Polyclonal inhibitory antisera—rabbit Ab	IgG	β-D-galactofuranosyl units of surface glycoproteins	*Trypanosoma cruzi*	research study	[15]
Human serum monoclonal Ab (L-5-27)	-	suggested it recognises terminal α-Gal*p*-(1,3)-β-Gal*f* epitope	*Leishmania major*	research study	[16]
Rabbit serum	-	Gal*f*-containing LPPG & LPGG-like molecules	*Trypanosoma cruzi*	research study	[17]
Rat monoclonal Ab (EB-A2)	IgM	(1,5)-β-Gal*f*-containing epitope of the galactomannan molecule	*Aspergillus fumigatus*	research study; diagnostics (Platelia^®^ ELISA, Pastorex^®^ LAT)	[12,18,19,20,21,22]
Human serum (paracoccidioidomycosis patients)	-	Gal*f*-containing glycolipid	*Paracoccidioides brasiliensis*	research study	[23]
Mouse monoclonal Ab (MEST-1)	IgG3	β-Gal*f*-(1,6)-α-Man*p*-(1,3)-β-Man-(1,2)-Ins. of GIPL-1 antigen	*Paracoccidioides brasiliensis*	research study	[24,25]
Mouse monoclonal Ab (L10-1 & L99-13)	IgM	Gal*f* of the galactomannan molecule	*Aspergillus fumigatus*	research study (immunohistology)	[26]
Mouse monoclonal Ab (MAb476)	IgM	Gal*f* moiety of the galactomannan epitope	*Aspergillus fumigatus*	research study (urinary immunochromatographic assay)	[27]
Mouse monoclonal Ab (mAb JF5)	IgG3	mannoprotein antigen	*Aspergillus fumigatus*	research study (immunoPET/MR imaging)	[28]
Human monoclonal Ab (mAb JF5)	IgG3	(1,5)-β-Gal*f* epitope of mannoprotein antigen	*Aspergillus fumigatus*	research study (immunoPET/MR imaging)	[29]
Mouse monoclonal Ab (mAb AP3)	IgG1κ	oligo-[β-Gal*f*-(1,5)] & Gal*f* residues of *O*-linked glycans on GM	*Aspergillus fumigatus*	research study	[30]
Mouse monoclonal Ab (mAbs 7B8 & 8G4)	-	β-D-Gal*f*-(1,5)-[β-Gal*f*-(1,5)]_3_-α-D-Man*p*	*Aspergillus fumigatus*	research study	[31]

**Table 2:** Comparative summary of natural lectins interacting with different Gal*f*-containing glycan motifs.

Lectins				
Origin	Epitope Specificity	Target Organism	Purpose	References
Fimbrial lectins (*Streptococcus* sp.)	diverse carbohydrate specificity among which also internal Gal*f* linked β-(1,6) to β*-*Gal-(1,3)-αGalNAc or β*-*GalNAc-(1,3)-α-Gal	*Actynomices viscosus*/*naeslundii*	research study	[41]
Adhesins (bacteria)	diverse carbohydrate specificity, among which also Gal*f*-disaccharide-binding regions	*Streptococci*	research study	[42]
DC-SIGN lectin from human monocyte-derived dendritic cells	multiple glycan epitopes, among which also Gal*f*-coated gold nanoparticles	Gal*f*-coated gold nanoparticles	research study	[43]
Human intelectin-1 (hIntL; hIntL-1/hINTL1); omentin; intestinal lactoferrin receptor; endothelial lectin HL-1	multiple glycan epitopes; arabinogalactan D-galactofuranosyl residues; Gal*f*-carrying column resins	Microbes; *Nocardia*	research study	[32,33,34,35]

**Table 3:** Codon changes and respective amino acid changes introduced at 464 positions in the wild-type Gal*f*-ase amino acid sequence by site-directed mutagenesis. Color indicates the mutation.

Mutant Variant	Codon Change	Amino Acid Change
E464A	GAG → GCG	Glu (E) → Ala (A)
E464**S**	GAG → AGC	Glu (E) → Ser (S)
E464**C**	GAG → TGC	Glu (E) → Cys (C)
E464**Q**	GAG → CAG	Glu (E) → Gln (Q)

**Table 4:** Comparison of Michaelis–Menten kinetics constants for hydrolysis of β-D-Gal*f* by E464X Gal*f*-ases mutant variants. Color indicates the mutation.

Enzyme	*K*_M_ (mM)	k_cat_/(min^−1^)	k_cat_/*K*_M_ (min^−1^.mM^−1^)
E464	0.25	213	852
E464**S**	0.036	>0.01	0.27
E464**C**	0.17	0.1	0.58
E464**Q**	0.166	2.5	15

**Table 5:** Comparison of BC_50_ of Gal*f*NGP towards different Gal*f*-ase-binding proteins and their respective values.

Proteins	BC_50_ (Gal*f*NGP/μM)
hIntL-1	0.016 +/− 0.004
Wild-type Gal*f*-ase	0.024 +/− 0.008
Gal*f*NeoLect	0.016 +/− 0.008

**Table 6:** IC_50_ of inhibitors towards wild-type Gal*f*-ase and Gal*f*NeoLect, expressed in mM.

Proteins	Gal*f*-N_3_	Gal*f*-Thioaryl	*p*NP-β-D-Gal*f*
Gal*f*NeoLect	1.39 +/− 0.8	0.15 +/− 0.04	1.14 +/− 1.0
Gal*f*-ase	0.59 +/− 0.2	0.18 +/− 0.04	0.56 +/− 0.2

**Table 7:** Primers used for site-directed mutagenesis.

Gene Mutation	Primer Length/bp	Primer Orientation	Primer Sequence (5′ → 3′) *^a^*
E464Q	25	Forward	GTCAACCAGAACCAGGGCTGGGGCC
		Reverse	GGCCCCAGCCCTGGTTCTGGTTGAC
E464A	27	Forward	CTGGCCCCAGCCCGCGTTCTGGTTGAC
		Reverse	GTCAACCAGAACGCGGGCTGGGGCCAG
E464C	35	Forward	CGTACTGGCCCCAGCCGCAGTTCTGGTTGACCCAC
		Reverse	GTGGGTCAACCAGAACTGCGGCTGGGGCCAGTACG
E464S	35	Forward	CGTACTGGCCCCAGCCGCTGTTCTGGTTGACCCAC
		Reverse	GTGGGTCAACCAGAACAGCGGCTGGGGCCAGTACG

*^a^* Underlined sequences indicate the substituted codons.

## Data Availability

Data are contained within the article or Supplementary Materials.

## Supplementary Materials

The following supporting information can be downloaded at: https://www.mdpi.com/article/10.3390/ijms25094826/s1.
