# Supplementary material for: Galf-Specific Neolectins: Towards Promising Diagnostic Tools
Source: Int J Mol Sci. 2024 Apr 28;25(9):4826. doi: 10.3390/ijms25094826 (PMC11084152; doi:10.3390/ijms25094826)
Supplement: Supplementary file 1 [file ijms-25-04826-s001.zip › ijms-2924657-supplementary.pdf]

Supplemental material

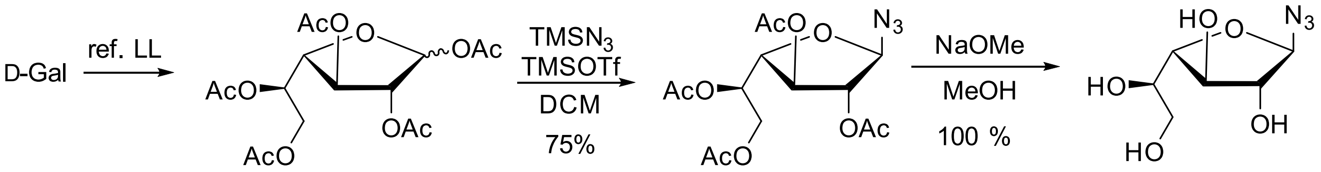

Figure S1. Schematic representation of synthetic pathway to Gal*f*-N_3 [49].

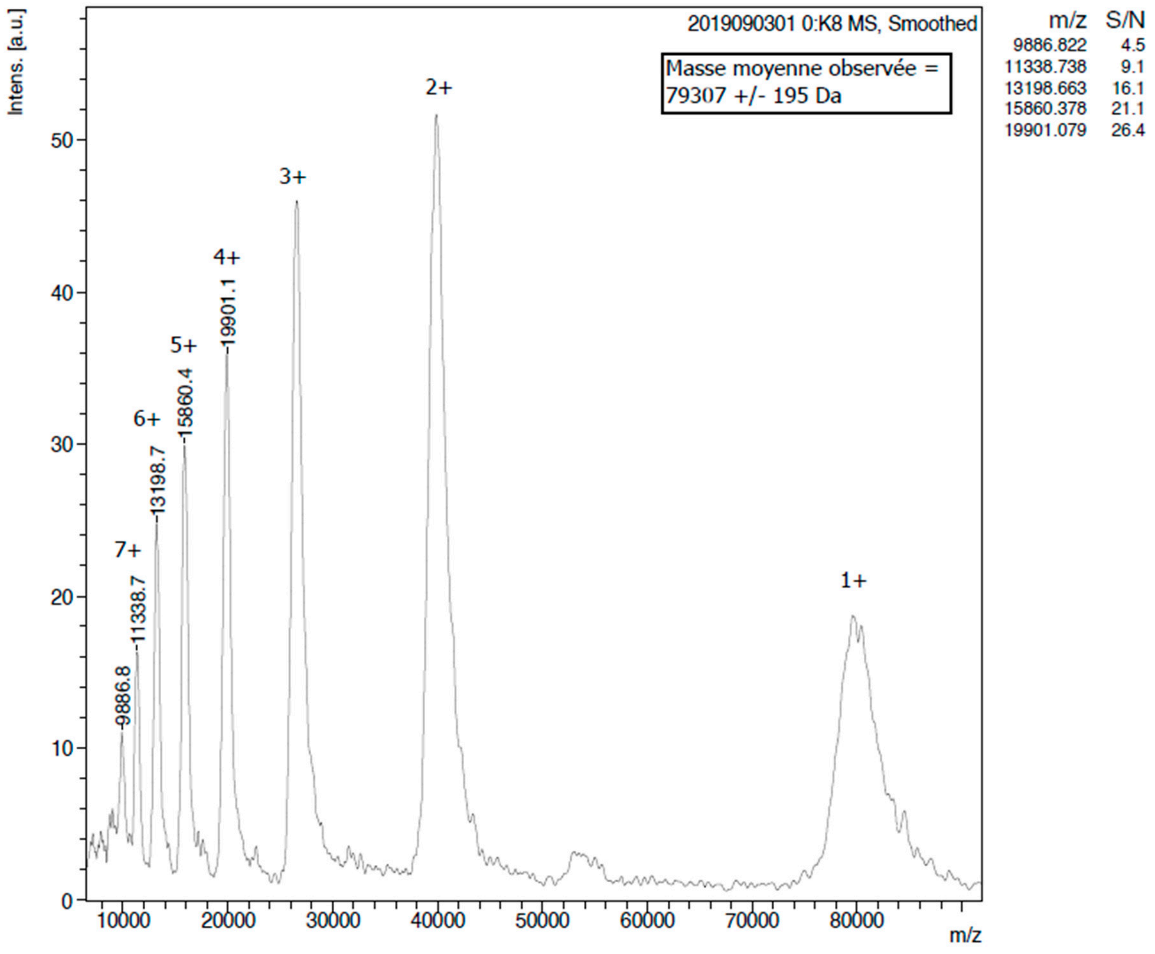

Figure S2A. MALDI-TOF result of Gal*f*NGP. Number of Gal*f*-units was calculated as follows: ($MW_{GalfNGP}$ - $MW_{BSA-alkyne}$)/MW_{GalfN3}. MW = Molecular weight. According to this formula, the number of Gal*f* on Gal*f*NGP is equal to 35.

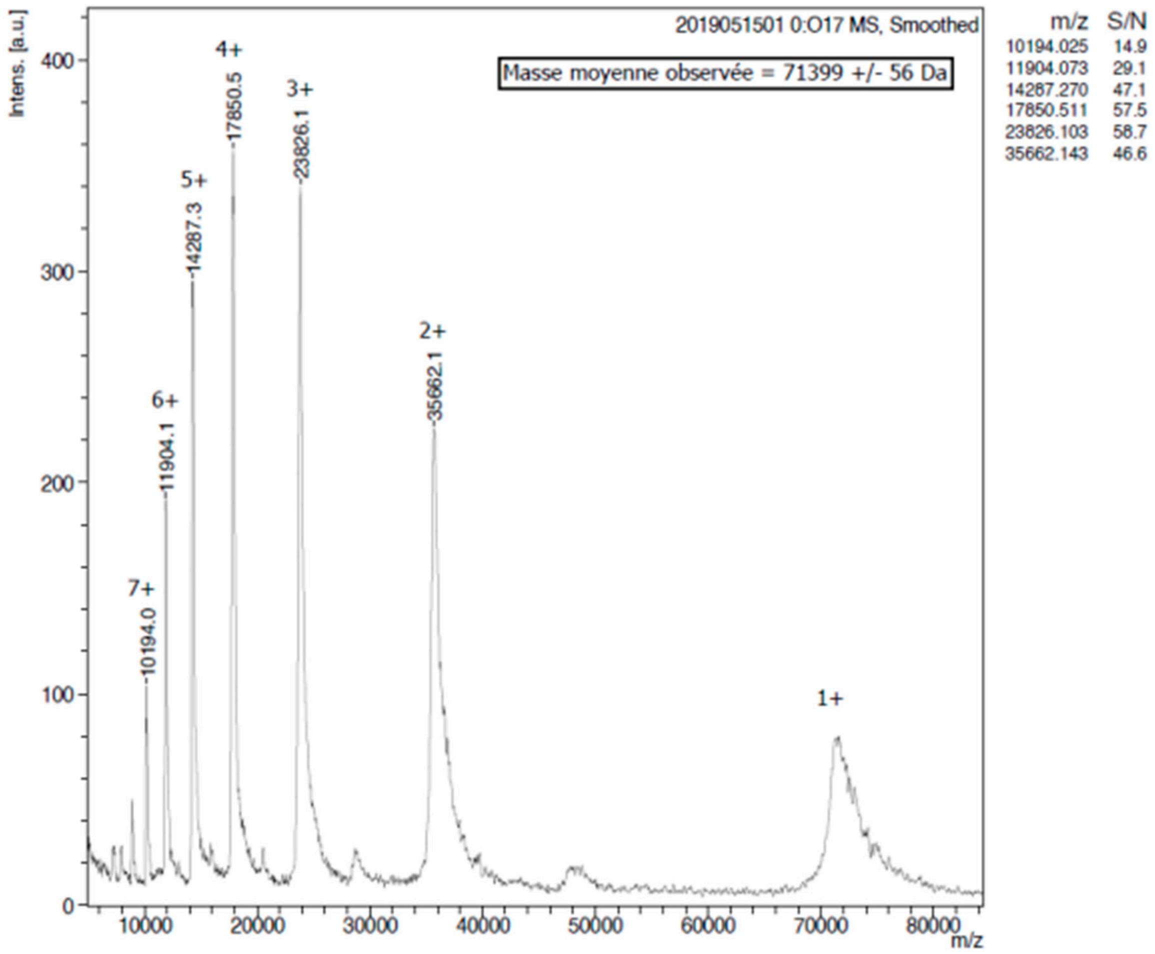

Figure S2B. MALDI-–TOF result of BSA-alcyne.

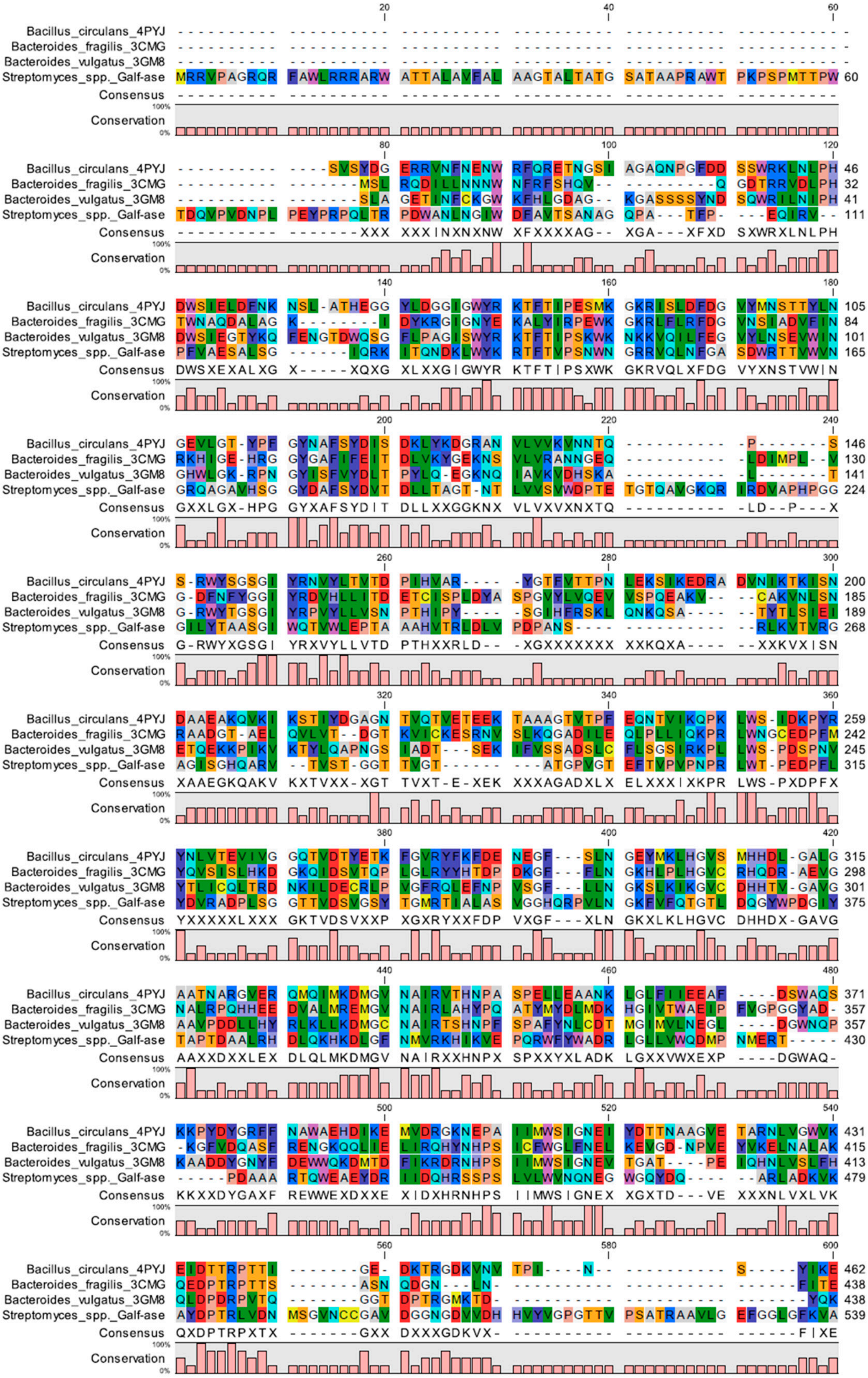

Bacillus_circulans_4PYJ
Bacteroides_fragilis_3CMG
Bacteroides_vulgatus_3GM8
Streptomyces_spp._Galf-ase
Consensus
Conservation

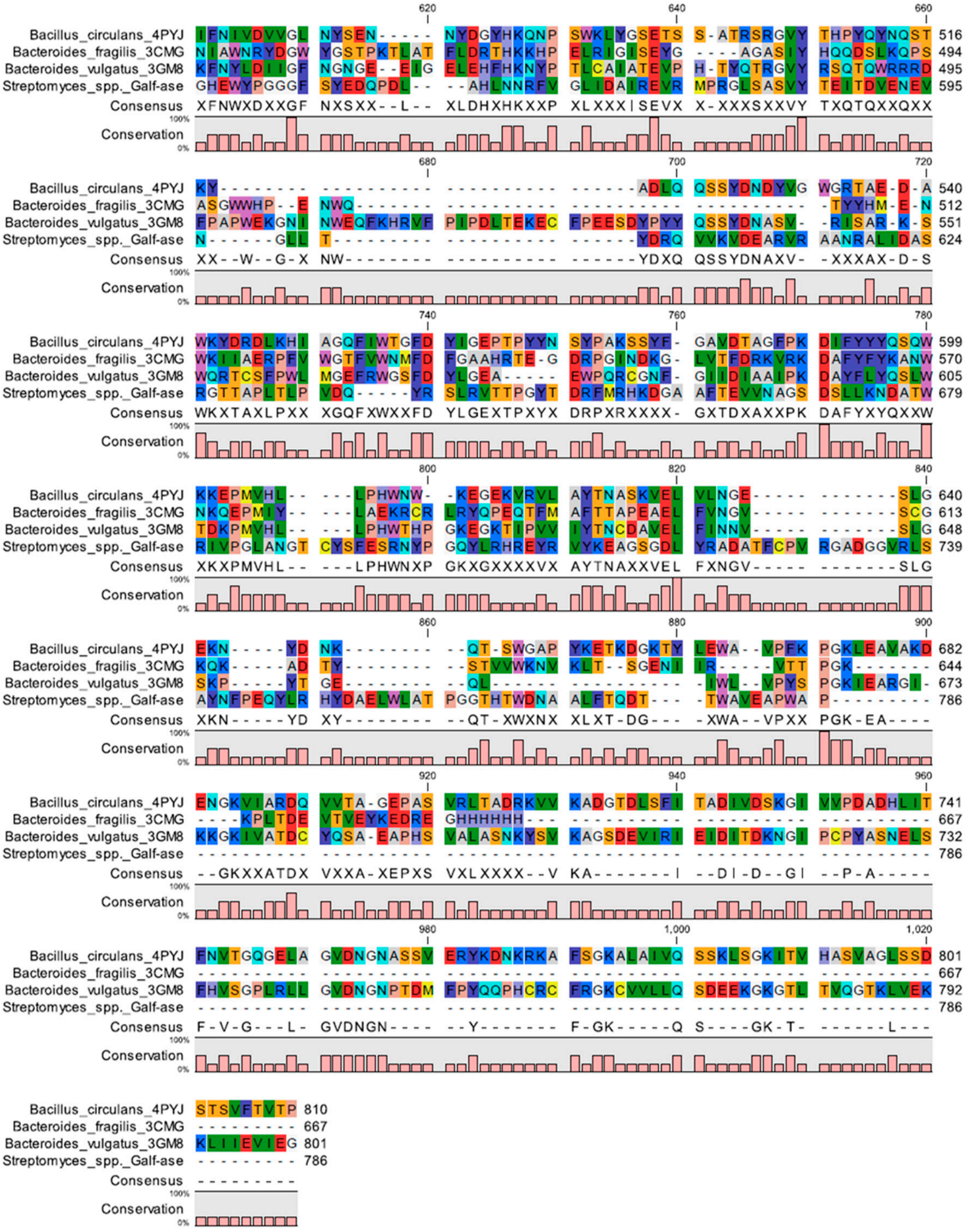

Figure S3. Alignment of complete ORF Gal*f*-ase (*Streptomyces spp.*) amino acid sequence and of its homologs.

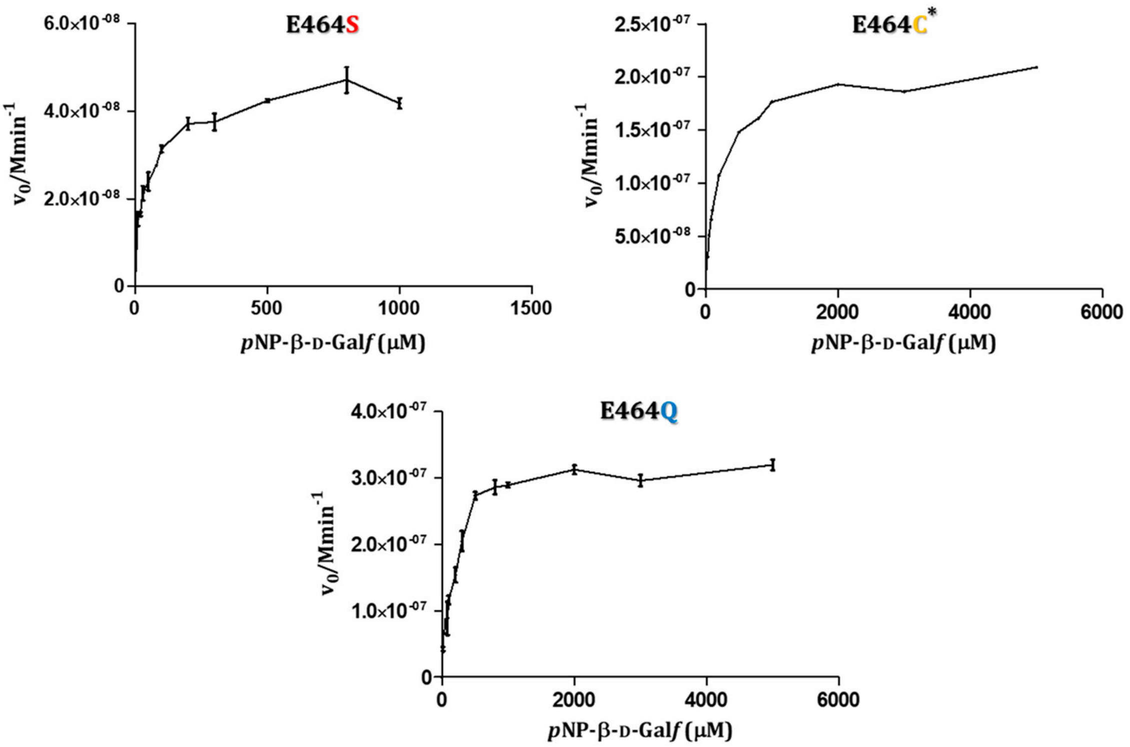

Figure S4. Michaelis–Menten plot of *p*NP-β-D-Gal*f* hydrolysis reaction catalysed by Gal*f*-ase E464S, E464C, and E464Q mutant variants. Mean values and SD error bars were calculated from three independent experiments (*except E464C) and from a single protein preparation.

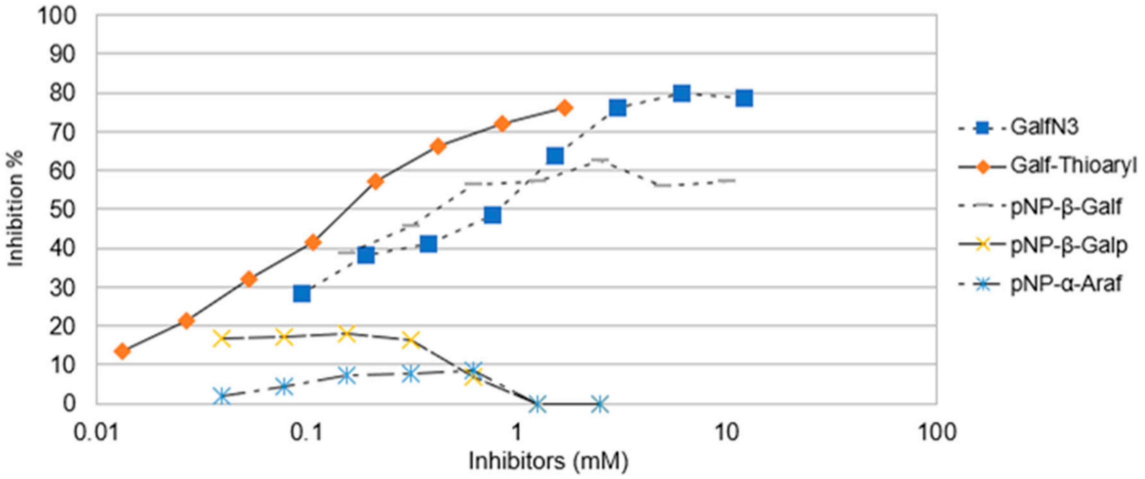

Figure S5. Inhibition profile of *p*NP-monosaccharides, Gal*f*-N_3, and Gal*f*-thioaryl with Gal*f*NeoLect. Biotinylated Gal*f*NGP (C=2μg/mL) was used as a tracer.

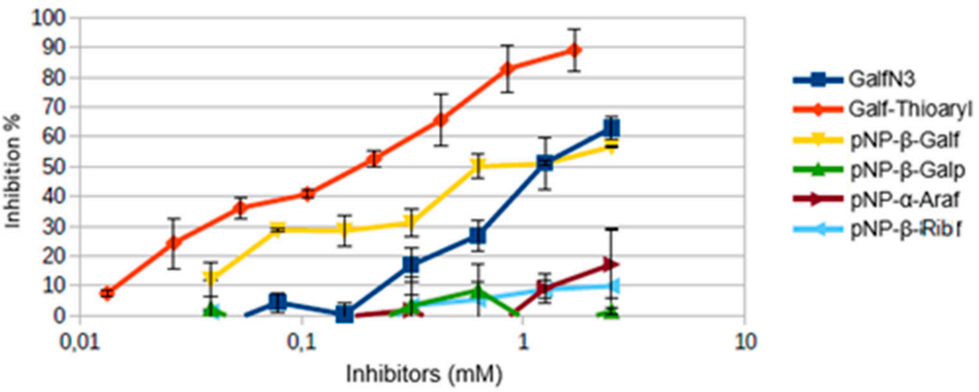

Figure S6. Inhibition profile of *p*NP-monosaccharides, Gal*f*-N_3, and Gal*f*-thioaryl with wild-type Gal*f*-ase. Biotinylated Gal*f*NGP (C=2µg/mL) was used as a tracer.

Table S1. Direct binding assays values of different NGPs (NeoGa, NeoF, NeoM, NeoaG and Gal*f*NGP, respectivelly functionnalised with α-D-Galactose, α-L-Fucose, α-D-Mannose, α-D-Glucose, and Gal*f*) to immobilized hIntL-1, wild-type Gal*f*-ase Gal*f*NeoLect.

**Proteins**	**NGPs**	**Concentration (µM)**	**Fluorescence intensity**
Wild-type Gal*f*-ase	NeoGa	0.5	2029
	NeoF	0.5	0
	NeoM	0.5	0
	NeoaG	0.5	2525
	Gal*f*NGP	0.13	2800
Gal*f*NeoLect	NeoGa	0.5	0
	NeoF	0.5	1652
	NeoM	0.5	1342
	NeoaG	0.5	918
	Gal*f*NGP	0.13	7691
hIntL-1	NeoGa	0.5	742
	NeoF	0.5	380
	NeoM	0.5	65
	NeoaG	0.5	125
	Gal*f*NGP	0.13	17560
